# A Retrospective Epidemiological Study of Mesothelioma in the United States (1999-2020) Based on Centers for Disease Control and Prevention Wide-Ranging Online Data for Epidemiologic Research (CDC WONDER) Data

**DOI:** 10.7759/cureus.95811

**Published:** 2025-10-31

**Authors:** Yashaswi Guntupalli, Bharat Vejandla, Tyler Evans, Piyush Ratan, Shivaashish Karumanchi Anantha Venkata Sai, Sai Prashanthi Varakala

**Affiliations:** 1 Internal Medicine, Sri Venkateswara Institute of Medical Sciences (SVIMS), Tirupati, IND; 2 Internal Medicine, All American Institute of Medical Sciences, Black River, JAM; 3 Internal Medicine, St. George’s University School of Medicine, St. George's, GRD; 4 Internal Medicine, Patna Medical College, Patna, IND; 5 Internal Medicine, Tagore Medical College and Hospital, Chennai, IND; 6 Internal Medicine, Osmania Medical College, Hyderabad, IND

**Keywords:** cdc-wonder, demography, disparity, incidence, mesothelioma, retrospective study

## Abstract

Introduction: Mesothelioma is a rare malignancy of the serous membranes, most commonly the pleura. Variations and demographic trends in mesothelioma incidence form the basis of this study.

Methodology: A retrospective study was conducted on August 30, 2024, using the Centers for Disease Control and Prevention Wide-Ranging Online Data for Epidemiologic Research database. The study analyzed age, gender, and race of mortality among mesothelioma patients in the United States from 1999 to 2020.

Results: There was a significant increase in the incidence of mesothelioma from 1999, peaking in 2011, followed by a gradual decline. Incidence was higher among individuals aged ≥75 years, men, and the White population.

Conclusions: This study highlights the higher incidence of mesothelioma in individuals aged ≥75 years compared to younger age groups, in men compared to women, and in White populations compared to other races. Overall, the incidence increased from 1999 to 2010, peaked in 2011, and declined thereafter through 2020.

## Introduction

Mesothelioma is a rare malignancy of the serous membranes with a global incidence rate of 0.30 cases per 100,000 people [1]. It most commonly occurs in the pleura but can also be seen in the peritoneum, pericardium, and tunica vaginalis. The strongest risk factor for pleural mesothelioma is asbestos exposure, even for a relatively short duration of less than or equal to two years [2]. Nonetheless, germline mutations in BRCA1-associated protein-1, radiotherapy, and Simian Virus 40 have also been attributed to the development of mesothelioma [2,3]. The disease has a long latent period, which could last up to 30-40 years from the first exposure to asbestos to the onset. The disease carries a grim prognosis with a median estimated survival of 8-14 months and a five-year relative survival rate of 12%, which is significantly lower than the five-year survival rate of 69.1% for all cancers [4].

National variations and trends in mesothelioma incidence exist, with various demographic groups affected differently. Gender differences in mesothelioma suggest that it is more common in men due to occupational exposure, such as mining, shipbuilding, and construction [5]. In recent decades, mesothelioma cases among women have shown an upward trend, likely linked to environmental asbestos exposure. Furthermore, racial and ethnic differences are evident, with White populations demonstrating comparatively higher incidence rates than other groups. The disease most often presents in the fifth and sixth decades of life, with a median age at diagnosis of approximately 74 years [6,7].

Recognizing these differences is important for developing focused public health measures and preventive strategies to meet the ongoing challenges. The present study examines temporal trends and demographic variations in mesothelioma incidence in the United States among individuals aged 15 years and older between 1999 and 2020, using data from the Centers for Disease Control and Prevention Wide-Ranging Online Data for Epidemiologic Research (CDC WONDER) database [8].

The present study aimed to analyze temporal trends in mesothelioma incidence in the United States from 1999 to 2020 using the CDC WONDER database. The primary objective was to assess changes in incidence rates over time, while the secondary objective was to evaluate demographic variations in incidence according to age, sex, and race. By examining these patterns, the study seeks to provide a comprehensive population-level overview of mesothelioma epidemiology and highlight groups that may benefit from targeted prevention and screening strategies.

## Materials and methods

A retrospective study was conducted using the CDC WONDER database, a publicly available resource that provides deidentified population-based data on a range of health outcomes, including cancer statistics [8]. Data were extracted on September 2, 2024, from the Cancer Incidence 1999-2020 dataset. This database includes cancer incidence data compiled from state cancer registries that participate in the National Program of Cancer Registries and the Surveillance, Epidemiology, and End Results (SEER) Program. Since the CDC WONDER database contains deidentified and publicly accessible data, the study was classified as non-human participant research and did not require ethics committee approval [8].

The study analyzed cases where mesothelioma was listed as the primary cancer site. The inclusion criteria comprised all individuals aged 15 years or older diagnosed in the United States between 1999 and 2020. Data were retrieved using default interface filters in CDC WONDER for U.S. residents, both sexes, and all races. The dataset provides both crude case counts and age-adjusted incidence rates per 100,000 persons, standardized to the U.S. 2000 standard population. All analyses in this study were performed using these age-adjusted rates, which allow for valid comparisons across demographic groups and time periods.

Demographic variables included age group, sex, and race. Age was categorized into the following groups: <15, 15-24, 25-34, 35-44, 45-54, 55-64, 65-74, and ≥75 years. Race categories followed CDC WONDER definitions and included White, Black or African American, Asian or Pacific Islander, and American Indian or Alaska Native. A small combined group labeled Other races and Unknown was retained as provided by the database to maintain consistency and data stability, as separating these subgroups could result in suppressed or unstable estimates. Suppression of low-frequency data cells (<16 cases) was automatically performed by the CDC WONDER system to ensure confidentiality; no manual alterations or imputations were made.

After extraction, data were exported into Microsoft Excel (Microsoft Corporation, Redmond, WA) for organization and preliminary review. The statistical analysis was performed using R software, version 4.3.1 (R Core Team, Vienna, Austria). Descriptive statistics were calculated to summarize incidence across demographic subgroups. Temporal trends were evaluated using a simple linear regression model with annual age-adjusted incidence rates as the dependent variable and calendar year as the independent variable. The slope of the regression line represented the average annual change in incidence. Figures and visualizations were generated using the ggplot2 package (version 3.5.0) to depict long-term trends and demographic variations in mesothelioma incidence between 1999 and 2020.

## Results

From 1999 to 2020, the incidence of mesothelioma was 1.03 cases per 100,000 persons, with the highest incidence observed among the White population, particularly in men and individuals aged 75 years and older. A total of 7,062,806,912 individuals were included in the study population.

Table 1 shows the age, gender, and racial distribution of mesothelioma cases in the United States from 1999 to 2020. The highest crude rate per 100,000 was recorded in the 75 years and older age group (7.97 per 100,000), men (1.59 per 100,000), and the White population (1.21 per 100,000). These findings highlight significant demographic disparities in mesothelioma incidence.

**Table 1:** Demographic characteristics of mesothelioma patients

Variables	Population	Count, n (%)	Crude rate per 100,000
Age (years)			
15-24	984,207,074	138 (0.19%)	0.01
25-34	959,283,218	493 (0.68%)	0.05
35-44	973,812,431	1,355 (1.86%)	0.14
45-54	968,241,074	3,950 (5.43%)	0.41
55-64	808,129,415	10,803 (14.85%)	1.34
65-74	540,456,270	21,305 (29.28%)	3.94
≥75	435,628,239	34,721 (47.72%)	7.97
Gender			
Male	3,481,479,919	55,476 (76.23%)	1.59
Female	3,581,326,993	17,303 (23.77%)	0.48
Race			
American Indian or Alaskan Native	93,121,600	298 (0.41%)	0.32
Asian or Pacific Islander	398,633,095	1,093 (1.5%)	0.27
Black or African American	964,214,883	3,479 (4.78%)	0.36
White	5,606,837,334	67,612 (92.9%)	1.21
Other races	Not applicable	297 (0.41%)	Not applicable

**Figure 1:**
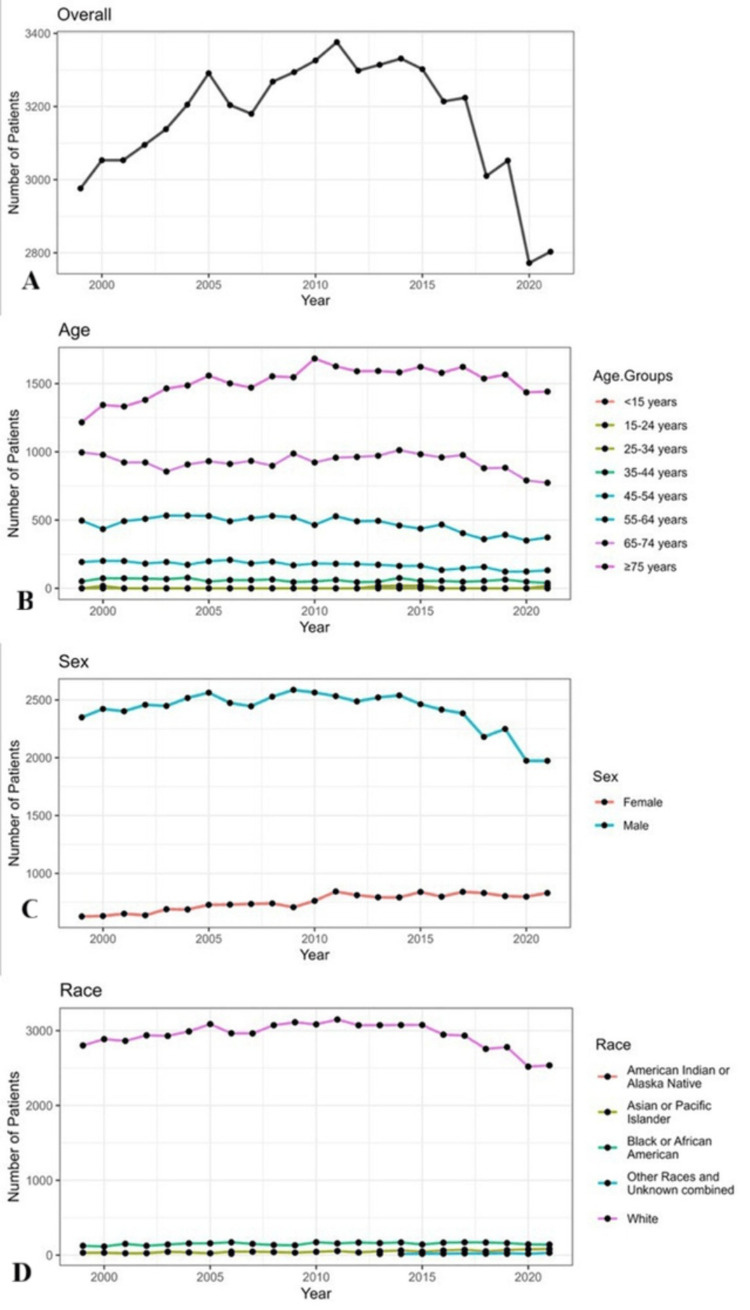
Trends in the incidence of mesothelioma in United States (A) Overall. (B) Age. (C) Sex. (D) Race

Figure 1 shows the trends in mesothelioma incidence in the United States. Over the 22-year period, the highest incidence of mesothelioma was recorded in 2011, followed by a gradual decline. The lowest incidence was recorded in 2020. Among age groups, individuals aged 75 years and older had the highest incidence, with a peak in 2010, whereas the under-15 age group had the lowest incidence throughout the study period.

Figure 1 also depicts the long-term trajectory of mesothelioma incidence over the years. The data reveal a steady rise in cases up to 2011, after which incidence began to decline. The incidence rate continued to increase among individuals aged 75 and older, while younger populations saw a downward trend. In men, the incidence started to decrease after 2015, whereas in women, it remained relatively stable.

Racial analysis showed that the highest incidence occurred among the White population, peaking in 2011 before gradually decreasing through 2020. Meanwhile, incidence rates in the Black or African American population remained consistently lower throughout the study period. These findings emphasize the importance of continued public health measures to mitigate mesothelioma risk, particularly among high-risk demographic groups.

## Discussion

In this retrospective original research study of mesothelioma incidence in the United States over a 22‐year period (1999-2020), we observed a crude incidence rate of 1.03 per 100,000 persons. Our findings indicate that the highest incidence was seen among individuals aged 75 years and older, in men, and within the White racial group. Temporal trends (as shown in Table 1 and Figure 1) revealed an overall rising incidence until 2011, followed by a gradual decline thereafter.

Mesothelioma, although a rare malignancy, remains an important public health issue due to its aggressive nature and its well-documented link to asbestos exposure. Studying cancer incidence patterns is crucial for identifying high‐risk groups and for assessing the effectiveness of preventive regulations and screening policies. The temporal trends observed in our study may signify both the long latency period of mesothelioma and the delayed effects of regulatory measures. Notably, legislative actions introduced in the 1970s and 1980s, which curtailed asbestos use in high-risk occupations, are likely contributors to the declining incidence observed after 2011 [1,2]. These measures have been credited with reducing asbestos exposure and, subsequently, mesothelioma risk. Moreover, our findings align with previous analyses using the SEER Program and global databases, which have similarly documented a peak in mesothelioma incidence followed by a downward trend owing to enhanced occupational safety standards and public health interventions [9,10].

A closer examination of demographic subgroups reveals important contrasts and similarities with other studies. Age remains a critical factor in mesothelioma risk. The marked increase in incidence among those aged 75 and older can be attributed to the disease's long latency period and the cumulative effects of asbestos exposure over a lifetime. This pattern is consistent with prior findings demonstrating that the burden of mesothelioma shifts toward older populations, reflecting historical exposures [7]. Gender differences are also evident. Our study reaffirms that men, likely due to their higher likelihood of employment in industries with substantial asbestos exposure, exhibit significantly higher incidence rates compared to women. However, our temporal analysis revealed a slight increase in mesothelioma cases among women beginning in 2011, with a plateau after 2015. This trend could be multifactorial, reflecting evolving exposure patterns (including environmental and secondary exposures), variations in diagnostic practices, or a lag in exposure effects unique to female populations. In contrast, the steady decline observed in male cases after 2015 suggests that regulatory measures may have had a more immediate impact in traditionally high-exposure settings [11]. Racial disparities further complicate the epidemiologic landscape; the highest incidence among the White population could be influenced by differential occupational exposures, socioeconomic factors, and access to healthcare services. Although similar trends have been reported in other regions of the United States and internationally, the interplay between race and environmental or occupational risk factors warrants further investigation to elucidate potential genetic or social determinants [5,12].

Looking ahead, several avenues for future work emerge from our study. First, there is a need for policy-driven strategies to enhance screening and early diagnosis among high-risk groups. Given that older adults, men, and White individuals demonstrate higher crude rates, targeted screening programs could facilitate early intervention and improve clinical outcomes. Furthermore, public health policies that continue to limit asbestos exposure, combined with community awareness initiatives about environmental risks, may help mitigate further increases in incidence, especially in populations where a slight upward trend (as observed among women) has been detected. Future studies would benefit from integrating more detailed clinical data, including information on tumor stage, grade, and patient outcomes, to better tailor both preventive measures and treatment protocols [13]. The development of comprehensive registries that capture data beyond initial diagnosis could be instrumental in guiding resource allocation and clinical decision-making [14-16].

Despite these strengths, our study is subject to several limitations. First, the most recent data (2021-2023) were not included, primarily because the COVID-19 pandemic disrupted data collection and reporting on the CDC WONDER platform. This gap limits our ability to assess whether the observed decline has continued or whether new patterns have emerged. Additionally, the lack of detailed information on tumor stage, grade, and long-term outcomes restricts our ability to correlate incidence trends with disease severity and survival. These limitations underscore the need for ongoing surveillance and more granular data collection in future research.

In summary, our study contributes valuable insights into the epidemiology of mesothelioma in the United States. By highlighting significant demographic variations and temporal trends, our findings not only corroborate previous research but also emphasize the importance of sustained regulatory and public health efforts. The observed decline in overall incidence following a peak in 2011 appears to be partially attributable to historical legislative measures restricting asbestos use. However, the nuanced trends, such as the plateau in female cases after 2015, suggest that additional factors may be influencing these patterns. Continued research that integrates detailed clinical data with robust environmental and demographic analyses will be essential for refining screening strategies, guiding policy interventions, and ultimately reducing the burden of this challenging disease.

Limitations

The main limitations of this study stem from the dataset used. The most recent data (2021-2023) are unavailable on the CDC WONDER website, largely due to disruptions in data collection during the COVID-19 pandemic [13]. Another limitation is the lack of detailed information on cancer stage, grade, and outcomes, which would have helped in better understanding incidence patterns and prognosis across populations.

## Conclusions

The crude rate per 100,000 of mesothelioma from 1999 to 2020 was 1.03. The rate was notably highest among individuals aged 75 years and older, at 7.97, followed by those aged 65-74 years, with a rate of 3.94. The incidence was significantly higher among men, with a crude rate of 1.59, compared to women at 0.48. Racial disparities were also evident, with White individuals having the highest crude rate of 1.21, while other races, such as Asian or Pacific Islanders and Black or African American populations, exhibited lower rates of 0.27 and 0.36, respectively. The temporal trends reveal that mesothelioma incidence increases markedly with age and is more prevalent in men and White populations.

Further research should be directed at understanding the stage or grade at diagnosis and the treatments assigned, as well as evaluating patient outcomes. Focusing on these aspects can help create policies for early identification and targeted screening, particularly in high-risk groups like the elderly and men. Moreover, improving treatment protocols and outcome monitoring could lead to better survival rates and quality of life for mesothelioma patients.
